# Single cell genomics indicates horizontal gene transfer and viral infections in a deep subsurface Firmicutes population

**DOI:** 10.3389/fmicb.2015.00349

**Published:** 2015-04-22

**Authors:** Jessica M. Labonté, Erin K. Field, Maggie Lau, Dylan Chivian, Esta Van Heerden, K. Eric Wommack, Thomas L. Kieft, Tullis C. Onstott, Ramunas Stepanauskas

**Affiliations:** ^1^Bigelow Laboratory for Ocean SciencesEast Boothbay, ME, USA; ^2^Department of Geosciences, Princeton UniversityPrinceton, NJ, USA; ^3^Lawrence Berkeley National LaboratoryBerkeley, CA, USA; ^4^Department of Microbial, Biochemical and Food Biotechnology, University of the Free StateBloemfontein, South Africa; ^5^Department of Plant and Soil Sciences, University of DelawareNewark, DE, USA; ^6^Department of Biology, New Mexico Institute of Mining and TechnologySocorro, NM, USA

**Keywords:** single cell genomics, *Desulforudis*, evolution, horizontal gene transfer (HGT), transposases, transposable phages, population genomics, terrestrial deep subsurface

## Abstract

A major fraction of Earth's prokaryotic biomass dwells in the deep subsurface, where cellular abundances per volume of sample are lower, metabolism is slower, and generation times are longer than those in surface terrestrial and marine environments. How these conditions impact biotic interactions and evolutionary processes is largely unknown. Here we employed single cell genomics to analyze cell-to-cell genome content variability and signatures of horizontal gene transfer (HGT) and viral infections in five cells of *Candidatus Desulforudis audaxviator*, which were collected from a 3 km-deep fracture water in the 2.9 Ga-old Witwatersrand Basin of South Africa. Between 0 and 32% of genes recovered from single cells were not present in the original, metagenomic assembly of *Desulforudis*, which was obtained from a neighboring subsurface fracture. We found a transposable prophage, a retron, multiple clustered regularly interspaced short palindromic repeats (CRISPRs) and restriction-modification systems, and an unusually high frequency of transposases in the analyzed single cell genomes. This indicates that recombination, HGT and viral infections are prevalent evolutionary events in the studied population of microorganisms inhabiting a highly stable deep subsurface environment.

## Introduction

Deep subsurface microorganisms constitute a significant fraction of the living biomass on our planet (Whitman et al., 1998; Kallmeyer et al., 2012), but our knowledge of these microorganisms remains very limited, due to the difficulties in accessing these environments without introducing microbial contamination and to our inabilities to isolate the indigenous microbiota through cultivation (Colwell and D'Hondt, 2013). Some deep subsurface fracture communities have been physically isolated from other habitable environments for up to tens of millions of years and are characterized by extreme energy limitation. Existing data suggest that deep subsurface microbial communities differ from those of surface environments in taxonomic composition, scarce energy sources, and energy production affected by a complete lack of light (Stevens and McKinley, 1995; Lin et al., 2006). They often have extremely low metabolic rates and generation times that span hundreds to thousands of years (Phelps et al., 1994; Jørgensen and D'Hondt, 2006; Lin et al., 2006; Onstott et al., 2014).

The deep fracture water of the Kaapvaal Craton, South Africa is a mixture of ancient hydrothermal fluid and meteoric water (Lippmann et al., 2003; Onstott et al., 2006; Lippmann-Pipke et al., 2011) that contains abiogenic hydrocarbons and H_2_-rich gasses (Ward et al., 2004; Lollar et al., 2006, 2008, 2014) and harbors unusual microbial communities and metazoans (Takai et al., 2001a,b; Moser et al., 2005b; Borgonie et al., 2011). A composite genome assembly of an indigenous firmicute *Candidatus Desulforudis audaxviator* MP104C was obtained from metagenomic reads from fracture water collected from a 2.8 km-deep borehole in the Mponeng gold mine (Chivian et al., 2008). The gene content of MP104C revealed a chemotactic sulfate-reducing bacterium capable of fixing N_2_ and CO_2_ while utilizing the chemical products of water radiolysis as its ultimate energy source (Chivian et al., 2008). Some of its key metabolic features, such as N_2_ fixation, cobalamin synthesis, and sulfite reduction appear to be products of ancient horizontal gene transfer (HGT) events from other bacteria and archaea. The presence of multiple transposases and CRISPRs in the MP104C genome provided evidence of HGT and interactions with phages. Surprisingly, >99.9% of the metagenomic reads from the Mponeng sample mapped to the composite genome assembly, and only 32 positions in the 2.35 Mbp genome contained single nucleotide polymorphisms. This indicated a unique, one-species ecosystem with one of the lowest reported rates of mutation. Ribosomal RNA sequences that are near-identical to MP104C have been detected in many subsurface sites outside the Witwatersrand Basin, including in the Fennoscandian Shield in Finland (Itävaara et al., 2011), a deep saline geothermal aquifer in Germany (Lerm et al., 2013), and the Juan de Fuca crustal basalt fluids (Jungbluth et al., 2013). This suggests that *D. audaxviator* is highly adapted to conditions of the immense deep subsurface environment and has a cosmopolitan distribution. However, to the best of our knowledge, 16S rRNA sequences have remained the only type of information about *Desulforudis* from locations outside the Mponeng fracture, leaving its genetic diversity and associated evolutionary and ecological implications largely unknown.

Single cell genomics (SCG) is a novel technology offering recovery of genomic information from individual, uncultivated cells (Stepanauskas, 2012). SCG provides quantitative information of genomic variability in natural microbial communities, allowing for the study of gene exchange among cells and genome rearrangements within a cell. Such information is hard to obtain using other methods, such as metagenomics, where genome assemblies are consensuses from a multitude of cells that are assumed to be clonal (Dupont et al., 2012; Iverson et al., 2012; Narasingarao et al., 2012).

To better understand the evolutionary mechanisms of natural *Desulforudis* populations, we performed genomic sequencing of individual cells collected from a 3.14 km-deep borehole in the Tau Tona mine, which is located near the site where *Ca*. *D. audaxviator* MP104C was obtained. We sequenced five single amplified genomes (SAGs) carrying SSU rRNA gene sequences with >99.5% identity to MP104C. Our results show that, despite a highly stable environment and extremely low cell abundance, microbial populations still engage in horizontal gene transfer, a process usually believed to be a strategy for fast adaptations to changing environmental conditions.

## Materials and methods

### Field sample collection

For single cell genomics, water samples were collected from the borehole DPH5057-TT109-Bh1 in the Tau Tona gold mine, located in the north margin of the Witwatersrand Basin, near Carletonville, South Africa, at 3.14 km depth below the surface. This borehole penetrated ~25 m ahead of a tunnel that was advancing through the seismically-active, Pretorius Fault Zone (Heesakkers et al., 2011) when it struck fracture water. The low microbial abundance (8000 cells mL^−1^), elevated pH (8–9) and temperature (48–49°C), and high reductive potential (340–370 mV) in the collected Tau Tona fracture water were typical of the deep subsurface of the Kaapvaal Craton (Magnabosco et al., 2014); while the ^14^C content of the DIC suggested a subsurface residence time of ~21 kyr (Supplementary Table 1).

An acid-washed, autoclaved stainless steel manifold with multiple sampling ports was connected to the valve that had been installed on the borehole by the mine. Sampling ports had a valve to adjust water flow rate to meet various sampling requirements. The port was opened to flush out the water for ~5 min before any sampling activities were undertaken. Unfiltered borehole water was collected in a 50 mL Falcon tube and transported to the laboratory on ice. Within 6 h, 1 mL aliquots were transferred to cryovials containing glyTE cryoprotectant (5% glycerol and 1x TE buffer pH 8.0, final concentrations), mixed gently, and kept frozen at −80°C until processing.

### Single cell sorting, whole genome amplification, sequencing, and assembly

Single cell sorting, whole-genome amplification, PCR-amplification and sequencing of the small subunit ribosomal RNA (SSU rRNA) genes, as well as the shotgun sequencing and *de novo* assembly of the selected SAGs were performed at the Bigelow Laboratory Single Cell Genomics Center (scgc.bigelow.org), as described previously (Stepanauskas and Sieracki, 2007; Swan et al., 2011; Martinez-Garcia et al., 2012; Field et al., 2015). PCR-amplified SSU rRNA gene sequences (~800–900 bp) of SAGs were edited using Sequencher v4.7 (Gene Codes) and compared with previously deposited sequences using the RDP v10 Classifier (SSU rRNA) (Wang et al., 2007) and National Center for Biotechnology Information (NCBI) BLAST (Altschul et al., 1990) nucleotide database (nt). Using SINA (Pruesse et al., 2012), the SAG SSU rRNA gene sequences were aligned with sequences selected with the RDP Seqmatch (SSU rRNA gene sequences from isolates, ≥1200 bp of good quality) from the RDP pipeline (Cole et al., 2014). A maximum likelihood tree of the SAG SSU rRNA gene sequences (100 bootstrap replicates) was constructed using phyML v3.1 (Guindon et al., 2010) with the best model [GTR model with a gamma distribution (+G), estimated rates of variation among sites and a proportion of invariable sites (+I)], as determined with jModelTest 2 (Darriba et al., 2012). Five SAGs having SSU rRNA genes with more than 99.5% identity to that of *Ca*. *D. audaxviator* were selected for genomic sequencing.

### Comparison of genomes and identification of unique SAG genes

Average nucleotide identity (ANI) was calculated using JSpecies (Richter and Rosselló-Móra, 2009) with the ANIb parameters. Comparisons of putative proteins from SAG genes were performed with MUMmer using the promer algorithm (Delcher et al., 2002). The SAGs were compared to each other and to the metagenomic assembly *Ca*. *D. audaxviator* MP104C (GenBank accession number NC_010424). Contigs that were similar to the MP104C genome but contained gaps were manually inspected and aligned using the Geneious Aligner (Biomatters, Auckland, New Zealand) with a cost matrix of 70% similarity (5.0/-4.0) to identify the specific regions of putative horizontal gene transfer. Gene annotations from the Integrated Microbial Genomes (IMG) (Markowitz et al., 2009, 2012) pipeline (http://img.jgi.doe.gov) were used to identify transferred genes and possible modes of horizontal gene transfer (tRNA recombination, transposition and homologous recombination).

### Functional classification of proteins

The predicted genes were translated and used to search against the NCBI non-redundant database (nr). The protein functions were classified with the COG analyzer (Tatusov et al., 2000) in MEGAN5 (Huson et al., 2011), where each “gene” was considered a “read.” Transposases were identified using the IMG annotations, and classified with the ACLAME database (Leplae et al., 2004).

### Metagenomic fragment recruitment

Previously published metagenomes from boreholes in Tau Tona (Lau et al., 2014; Magnabosco et al., 2014), Mponeng (Chivian et al., 2008), and Masimong (Lau et al., 2014; Magnabosco et al., 2014) gold mines were used to look at the geographic distribution of *Desulforudis* (Supplementary Table 2). BLAST+ v2.2.28 (Camacho et al., 2009) was used to recruit metagenome reads to each SAG assembly using BLASTn with default parameter values, except for the following: -evalue 0.0001 -soft_masking true -lcase_masking -xdrop_gap 150. Fragment recruitment results, along with whole genome comparisons were displayed using Circos v0.66 (Krzywinski et al., 2009).

### Calculation of HGT rate

Protein sequences from each sequenced SAG were compared with the NCBI nr database using BLASTp with an *e*-value cut off of 0.001, and the results were searched for “paradoxical” best hits, i.e., best hits to homologs not in *Ca*. *D. audaxviator* MP104C, as in Koonin (2001). All automatically detected “paradoxical” best hits were manually checked to eliminate possible false positives.

### Phylogenetic analysis of the virion morphogenesis protein

Analysis of the gene content of the SAGs revealed a transposable phage in SAG AC-310-N13. Sequences similar to the virion morphogenesis protein of the phage were obtained via BLASTx against the NCBI non-redundant database (nr). Protein sequences were aligned using MUSCLE (Edgar, 2004), and the alignment was manually edited in Geneious (Kearse et al., 2012). Phylogeny was performed using phyML (Guindon et al., 2010) implemented in Geneious with 100 bootstrap replicates and the LG model with a gamma distribution (+G), estimated rates of variation among sites and a proportion of invariable sites (+I). Trees were viewed with FigTree (http://tree.bio.ed.ac.uk/software/figtree/).

### Accession numbers

The annotated genomes are available in the Integrated Microbial Genomes database (https://img.jgi.doe.gov) under the IMG Taxon ID numbers 2596583550 (AC-310-A06), 2596583551 (AC-310-E02), 2600254919 (AC-310-N13), 2596583553 (AC-310-O10), and 2596583554 (AC-310-P15). The unassembled reads of single amplified genomes are available through the SCGC public data portal, http://data.bigelow.org/~scgc/.

## Results and discussion

### Generation, identification, and genomic sequencing of single amplified genomes (SAGs)

**Figure 1:**
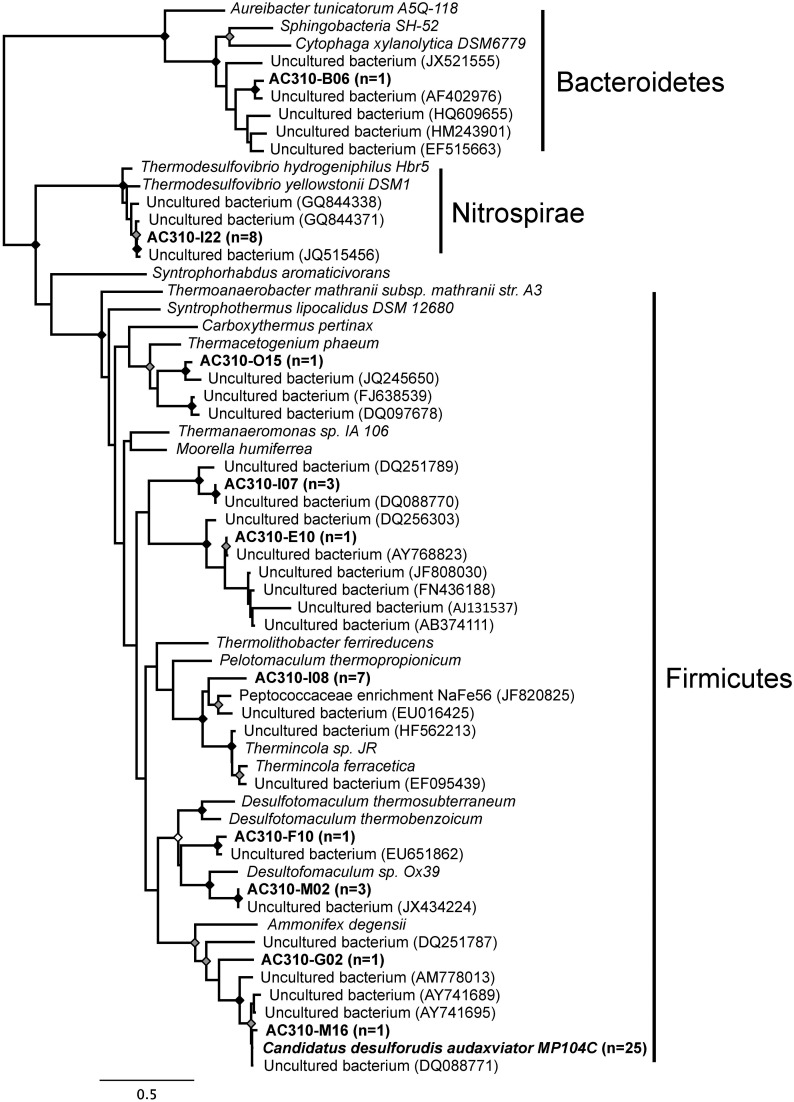
**Maximum likelihood phylogeny of the SSU rRNA gene sequences from SAGs (in bold) and closest relatives in GenBank**. The number of single cells with identical SSU rRNA gene sequences is indicated in parentheses. The tree was generated using maximum likelihood, with 100 bootstrap replicates, using the GTR model with a gamma distribution (+G), estimated rates of variation among sites and a proportion of invariable sites (+I). Bootstrap replicates 100%, ≥ 95%, and ≥85% are shown by black, gray, and white dots, respectively, at the nodes.

Small subunit rRNA gene sequences were obtained from 52 of the 315 (17%) multiple displacement amplification reactions containing individual cells. This percentage was slightly lower than 19–39% obtained from marine (Swan et al., 2011) and freshwater (Martinez-Garcia et al., 2012) bacterioplankton using the same techniques. The retrieved SSU rRNA gene sequences revealed SAGs belonging to Firmicutes (43 SAGs), Nitrospirae (8 SAGs) and Bacteroidetes (1 SAG) (Figure 1). The SSU rRNA genes formed 10 clusters with >99% sequence identity to each other and to sequences that were previously retrieved from diverse subsurface environments (Figure 1). Of the 52 identified SAGs, 25 contained SSU rRNA genes that were 99.5-100% identical to that of *Ca*. *D*. *audaxviator* MP104C. This shows that close relatives of *Desulforudis* inhabit multiple subsurface environments and can coexist with other microbial species, in support of prior, metagenomic evidence (Moser et al., 2003, 2005a; Lin et al., 2006).

**Figure 2:**
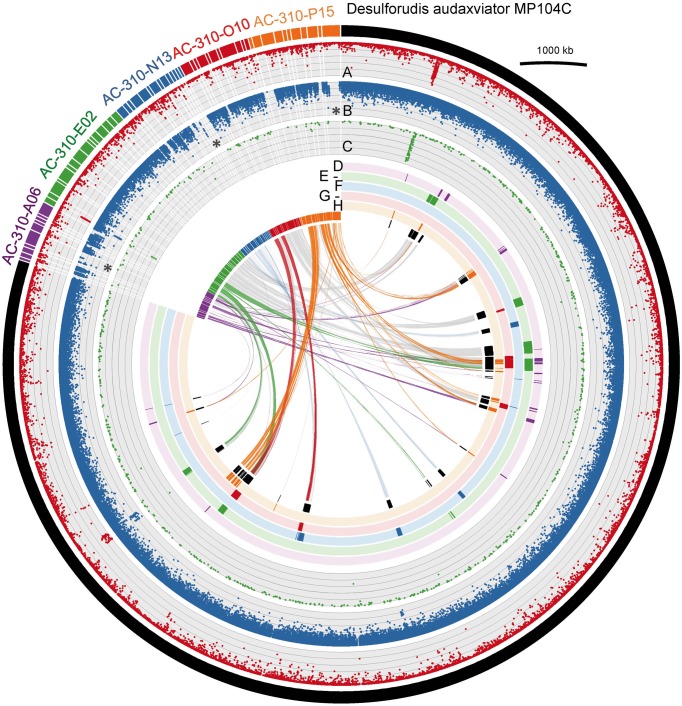
**Comparison of the sequenced SAGs AC-310-A06, AC-310-E02, AC-310-N13, AC-310-O10, and AC-310-P15 (colored outer circle segments) to the metagenomic assembly *Ca. D. audaxviator* MP104C (black outer circle segment)**. Circles A–D show results of the metagenomic fragment recruitment from Tau Tona, Mponeng and Masimong fracture samples, respectively. The six colored inner circles indicate MP104C genes recovered from each SAG. The inner-most circle (in black) indicates the presence of MP104C genes in any of the five SAGs. The links represent similarities based on MUMmer comparisons, using a 90% nucleic acid identity threshold. Asterisks indicate regions of low metagenomic fragment recruitment. Metagenomic fragment recruitment was performed with BLASTn, and hits with 75–100% DNA identity to references are displayed.

Of the five *Desulforudis* SAGs that were genomically sequenced, AC-310-E02 and AC-310-O10 had SSU rRNA genes that were 100% identical to MP104C, while AC-310-A06, AC-310-N13, and AC-310-P15 had SSU rRNA genes that were 99.5% identical to MP104C. Between 66 and 129 kbp of DNA sequence was recovered from each SAG in contigs larger than 2 kbp, corresponding to 4–8% of the expected genome size (Table 1). This SAG genome recovery was significantly lower (*p* < 0.001, *t*-test) than the 55% average genome recovery reported in a recent single cell genomics study of surface ocean bacterioplankton, which employed comparable laboratory and computational procedures (Swan et al., 2013). The unusually low genome recovery from *Desulforudis* SAGs may have been caused by multiple factors, such as incomplete cell lysis; chemical interactions of DNA with other molecules that prevented amplification; or inefficient multiple displacement amplification resulting from the high GC content (61%) of *D. audaxviator*. Another plausible explanation is that the analyzed *Desulforudis* cells were endospores or other resting stages that were difficult to lyse. Inside an endospore, DNA is protected by small acid-soluble proteins (Setlow, 1995) that may not be denaturated with the alkaline lysis of the cells used in this study. Further support for this hypothesis is provided by the presence of sporulation genes in *Ca*. *D. audaxviator* MP104C (Chivian et al., 2008) and the unusually dim fluorescence of Tau Tona microbial cells with the SYTO-9 nucleic acid stain (Supplementary Figure 1), which is typical for hard-walled microbial resting stages (Setlow et al., 2002). We are not aware of studies of microbial sporulation in the Kaapvaal Craton or other terrestrial subsurface environments. However, about half of the microbial cells within deep marine sediments are reported to be in a dormant state (Lennon and Jones, 2011; Lomstein et al. 2012; Hoehler and Jørgensen 2013). Some regions of MP104C appear to be preferentially recovered in SAGs (inner circles in Figure 2). A possible explanation is that DNA follows a specific folding pattern inside cells or endospores, with only some genome regions being accessible to MDA. Another plausible explanation is preferential recovery of low %GC regions in SAGs. The lower %GC of the SAG assemblies (54–56% GC) relative to the 61% GC of the MP104C genome is consistent with the latter hypothesis.

**Table 1:** **Genomic sequence recovery from single cells of *Desulforudis***.

**Genome**	**# raw reads**	**Assembly length (bp)**	**Largest contig (bp)**	**# contigs**	**GC content (%)**	**# genes**	**Genome recovery (%)**
AC-310-A06	9,248,293	66,621	18,148	10	54.4	84	4.1
AC-310-E02	9,194,185	129,196	16,829	16	56.9	148	7.8
AC-310-N13	14,367,041	78,324	12,465	14	57.3	105	3.6
AC-310-O10	8,678,532	79,711	20,659	11	55.0	105	6.4
AC-310-P15	8,362,251	107,785	24,300	11	56.0	119	4.7

*Only contigs longer than 2000 bp were included in this analysis. The % genome recovery estimate is based on the assumption that genomes of all Desulforudis have the same length as MP104C, i.e., 2,349,476 bp (Chivian et al., 2008). NA, Not applicable*.

### Genome content variation among desulforudis cells

The average nucleotide identity (ANI) among the five sequenced SAGs and the MP104C genome ranged between 88.5 and 99.9% (Supplementary Table 3). This demonstrates a significant divergence of homologous genome regions among the analyzed *Desulforudis* cells, despite their 16S rRNA genes being ≥99.5% identical to each other. Interestingly, 10 SAG contigs (out of a total of 62) had no homology to MP104C (Supplementary Table 4). Another 22 SAG contigs contained both conserved and novel genome regions, when compared to MP104C (showcased as links in Figure 2, and Supplementary Figure 2). The significant recruitment of metagenomic fragments from Tau Tona but not Mponeng and Masimong by some of the SAG-specific genome regions demonstrated their geographic endemism (asterisks in Figure 2 and Supplementary Table 5).

**Figure 3:**
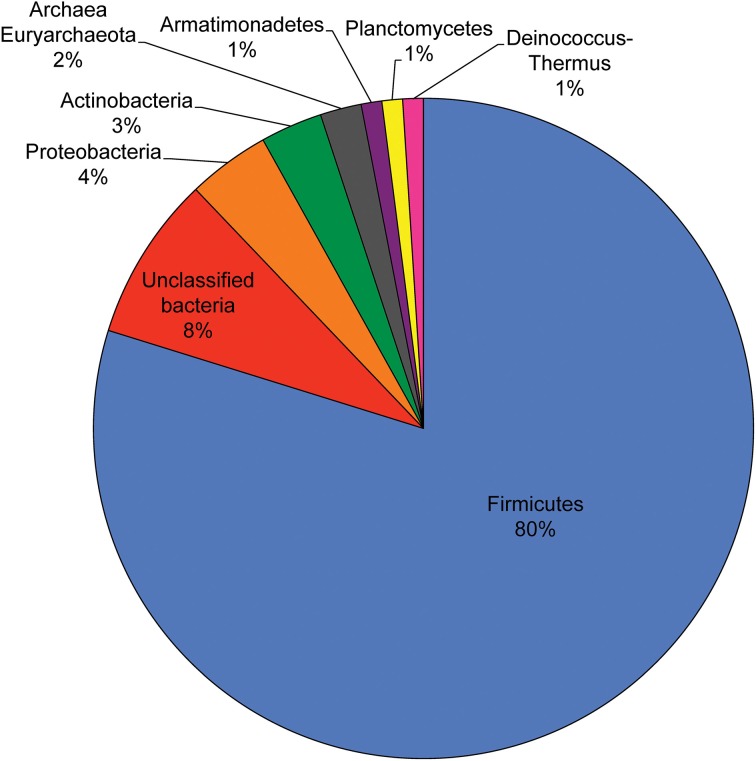
**Putative origin of the 99 genes that were found in SAGs but not in *Ca. D. audaxviator* MP104C, as determined by the “paradoxical” blast**.

Between 0 and 32% of genes found in the sequenced SAGs were absent in the *Ca. D. audaxviator* MP104C genome (Supplementary Table 6), suggesting that they are products of horizontal gene transfer (HGT). In other taxa of Firmicutes, such as *Bacillus subtilis* and *B. halodurans*, the percentage of newly acquired genes has been estimated to ~9%, while rates of up to 20% have been observed in Spirochaetes (Koonin, 2001). The majority of SAG genes (80%) with no homology to *Ca. D. audaxviator* MP104C were similar to genes found in other Firmicutes, such as *Desulfotomaculum* sp. and *Pelotomaculum* sp. (Figure 3). Interestingly, many of these novel regions were in close proximity to transposases (9 cases), tRNA (2 cases), recombinases (2 cases), CRISPRs (1 case), and a prophage (1 case), all of which are indicative of recombination and HGT (Ochman et al., 2000; Gogarten et al., 2002; Brüssow et al., 2004; Marraffini and Sontheimer, 2010) (Supplementary Figure 2, and Supplementary Table 7). These results indicate a high prevalence of HGT events among autochthonous bacterial populations of the deep subsurface, despite their extremely slow metabolism and generation times that can be hundreds to thousands of years (Phelps et al., 1994; Jørgensen and D'Hondt, 2006; Lin et al., 2006).

The recovered regions of the five sequenced SAGs encoded for 20 transposases, representing 3.6% of all genes (Supplementary Table 8). The MP104C genome encodes 35 transposases, accounting for only 1.5% of all genes, which is still above the 0.83% average found in all sequenced microbial genomes and metagenomes (Aziz et al., 2010). This suggests that transposition may be significant in the evolution of *Desulforudis* natural populations. Transposases are among the most abundant genes in nature (Aziz et al., 2010) and are best known for their role in the HGT of antibiotic resistance genes (Scott, 2002; Whittle et al., 2002) and the relocation of regulatory elements (Shapiro, 2010). The only other study of transposase genes in a subsurface environment that we are aware of, in the Lost City deep hydrothermal system, also found an unusually high abundance of these genes, with more than 8% of metagenomic shotgun reads having a significant similarity to transposases (Brazelton and Baross, 2009).

**Figure 4:**
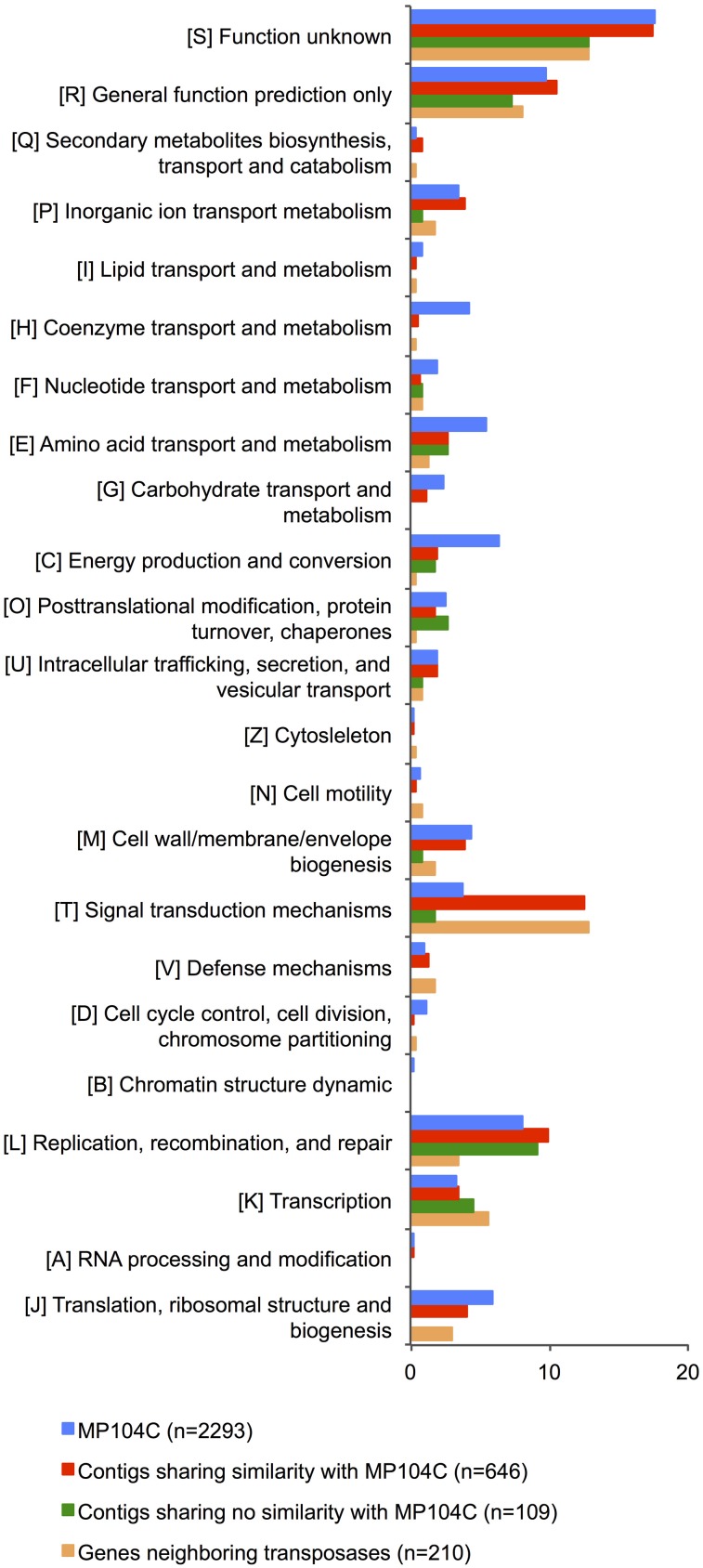
**COG category distribution (%) of (a) genes encoded by *Ca. D. audaxviator* MP104C (blue); (b) homologous genes encoded by SAGs (red); (c) non-homologous genes encoded by SAGs (green); and (d) genes neighboring transposase genes (orange)**.

To identify the genes that may have been acquired by transposition, we looked at neighborhoods of transposases on the SAG contigs and classified them based on their COG function (Supplementary Table 9). Most of the genes (57%) were not assigned or had no hits to the database. Intriguingly, most identified genes were involved in signal transduction (30% or the identified genes) and transcription/transcription regulation (11%) (Figure 4; Supplementary Table 9). This suggests that transposition in *Desulforudis* populations may be primarily involved in the evolution of regulation and communication rather than new metabolic capabilities.

The apparent prevalence of HGT in an environment with extremely low cell abundance and slow growth is puzzling. A plausible explanation may be provided by the possibility that biofilm-associated rather than free-living cells dominate subsurface microbiome (Taylor and Wirsen, 1999; Cozzarelli et al., 2007). Densely populated biofilms may provide microenvironments that enhance horizontal gene transfer (Molin and Tolker-Nielsen, 2003) and may serve as a source of cells and spores to the fracture water that was analyzed in this study. Further studies are required to determine the presence and role of biofilms in the Witwatersrand Basin.

### Signatures of desulforudis interactions with phages and mobile genetic elements

**Figure 5:**
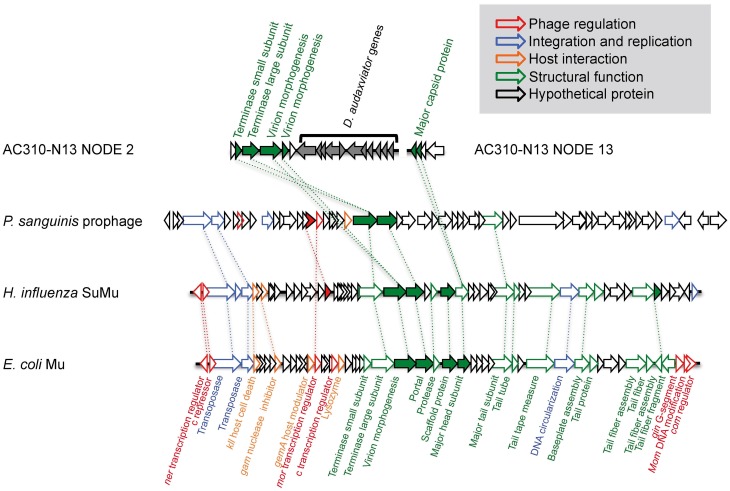
**Genomic organization of the partially assembled Mu-like transposable prophage in AC-310-N13 and its closest confirmed relatives**.

Two contigs in AC-310-N13 (NODE_2 and NODE_13) contain phage-like sequences with no homology to MP104C (Supplementary Table 4). The presence of genes encoding a virion morphogenesis protein, a major capsid protein, and a terminase protein that are distantly related to Mu-like phages, and the co-occurrence of bacteria- and phage-like genes on NODE_2 indicate a Mu-like transposable prophage (Figure 5). Transposable phages are believed to be temperate phages. They reproduce by transposition as part of their infectious cycle, in this way facilitating HGT and impacting the evolution of their hosts (Wang et al., 2004). Very few transposable phages have been isolated and sequenced to date. However, a recent re-analysis of genomic databases has revealed Mu-like prophages within the genomes of several Firmicutes and Proteobacteria (Toussaint, 2013). The virion morphogenesis protein in AC-310-N13 is distantly related to prophages in other subsurface bacteria and non-subsurface Firmicutes, indicating co-evolution of these phages and their hosts (Supplementary Figure 3). The low nutrient concentrations observed in the deep subsurface are expected to be unfavorable for lytic phage infections (Fuhrman, 1999). Therefore, lysogeny may be the preferred lifestyle for deep subsurface phages. At least one prior study has indicated the prevalence of lysogeny in deep hydrothermal vents (Williamson et al., 2008). A selective advantage of lysogenic phage-host systems may be the immunity induced by them against other phages (Canchaya et al., 2003). Prophages sometimes increase the fitness of their host through lysogenic conversion or the transfer of useful genetic information (Brüssow et al., 2004). To the best of our knowledge, our study is the first to demonstrate the presence of transposable phages in the deep subsurface and their interactions with indigenous microorganisms.

We detected a CRISPR and associated *cas* genes in AC-310-E02 (Supplementary Figure 4). The same loci were also present in the MP104C genome, with some differences in the CRISPR spacers. Three additional CRISPR loci were also present in MP104C. However, none of the identified CRISPR spacers were homologous to the Mu-like viral sequences in AC-310-N13. Furthermore, genes encoding a type III restriction-modification system and a restriction endonuclease were found in AC-310-E02, while a type I restriction modification protein was found in AC-310-N13. The presence of these phage defense mechanisms provides further evidence for the importance of phage-host interactions in the ultra-deep fractures of the Kaapvaal Craton. While several prior studies report microscopic observations of viral particles in terrestrial subsurface (Kyle et al., 2008), the prevalence of lysogeny in hydrothermal systems (Williamson et al., 2008; Engelhardt et al., 2013), and the presence of prophage-like sequences in subsurface cultured isolates (Coombs, 2009), our study may be the first to identify transposable phage interactions with their hosts in indigenous, predominant microorganisms of the deep terrestrial subsurface.

Remarkably, one of the SAG contigs with no homology to MP104C, AC-310-P15 NODE_5, carries a gene for a retron-type RNA-directed DNA polymerase. Retrons are small genetic elements that encode for a reverse transcriptase (RT) similar to the RT found in retroviruses and other retroelements. Retrons remain poorly characterized, but are found in many bacterial genomes and could play a role in the evolution and fitness of their host (Lampson et al., 2005; Simon and Zimmerly, 2008). To the best of our knowledge, this is the first record of retrons in deep subsurface environments.

## Conclusions

Our study confirms that close relatives (>99.5% identity of the 16S rRNA gene) of the firmicute *Candidatus Desulforudis audaxviator* inhabit multiple deep subsurface fractures of the Kaapvaal Craton in South Africa. Unexpectedly, we found that *D. audaxviator* coexists with other bacterial species and undergoes frequent horizontal gene transfer and viral infections. Our findings of a prophage, a retron, multiple CRISPRs and restriction-modification systems, and an unusually high frequency of transposases suggest that recombination, HGT and viral infections may play a role in the evolution of indigenous microorganisms in the deep subsurface, despite their extremely low cell abundance, slow metabolism and long generation times.

### Conflict of interest statement

The authors declare that the research was conducted in the absence of any commercial or financial relationships that could be construed as a potential conflict of interest.
