# Supplementary material for: Single cell genomics indicates horizontal gene transfer and viral infections in a deep subsurface Firmicutes population
Source: Front Microbiol. 2015 Apr 22;6:349. doi: 10.3389/fmicb.2015.00349 (PMC4406082; doi:10.3389/fmicb.2015.00349)
Supplement: Supplementary file 1 [file DataSheet1.PDF]

# SUPPLEMENTARY MATERIALS

## ***Single cell genomics indicates horizontal gene transfer and viral infections in a deep subsurface Firmicutes population***

Jessica M. Labonté, Erin K. Field, Maggie Lau, Dylan Chivian, Esta Van Heerden, K. Eric Wommack, Thomas L. Kieft, Tullis C. Onstott, and Ramunas Stepanauskas

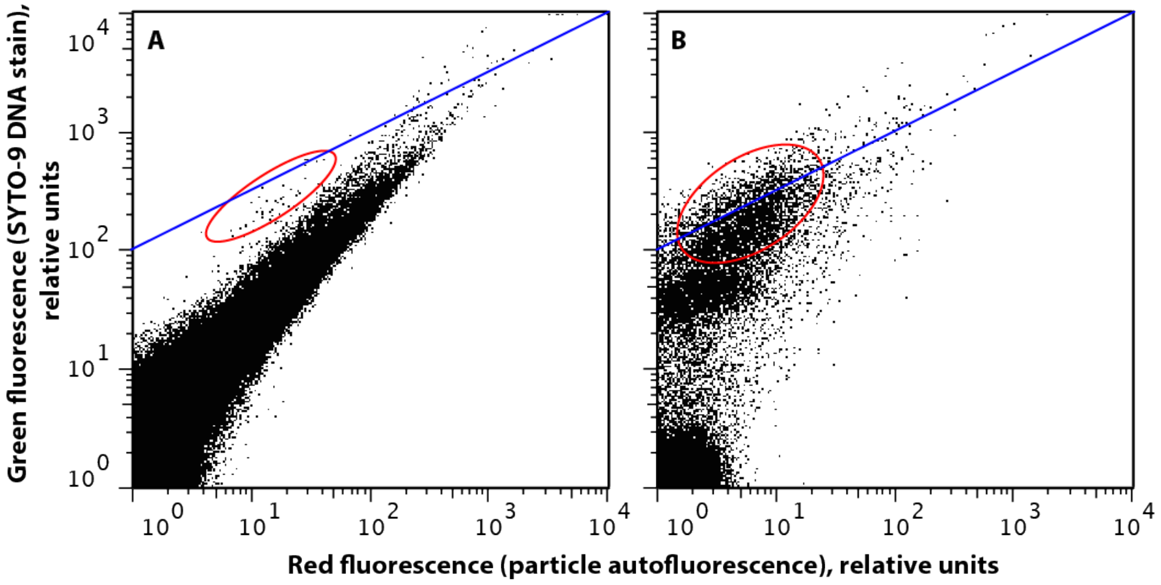


**Supplementary Figure 1:** Flow cytometric scattergrams of microbial communities from Tau Tona fracture TT109 (A) and the Gulf of Maine surface water (B). Red regions delineate prokaryote cells. Blue line was drawn to guide the comparison of fluorescence signals from the two microbial communities. Both samples were treated in the same way, as described in Materials and Methods of this paper (TT109 sample) and by Martinez-Garcia et al. (2012) (Gulf of Maine sample).

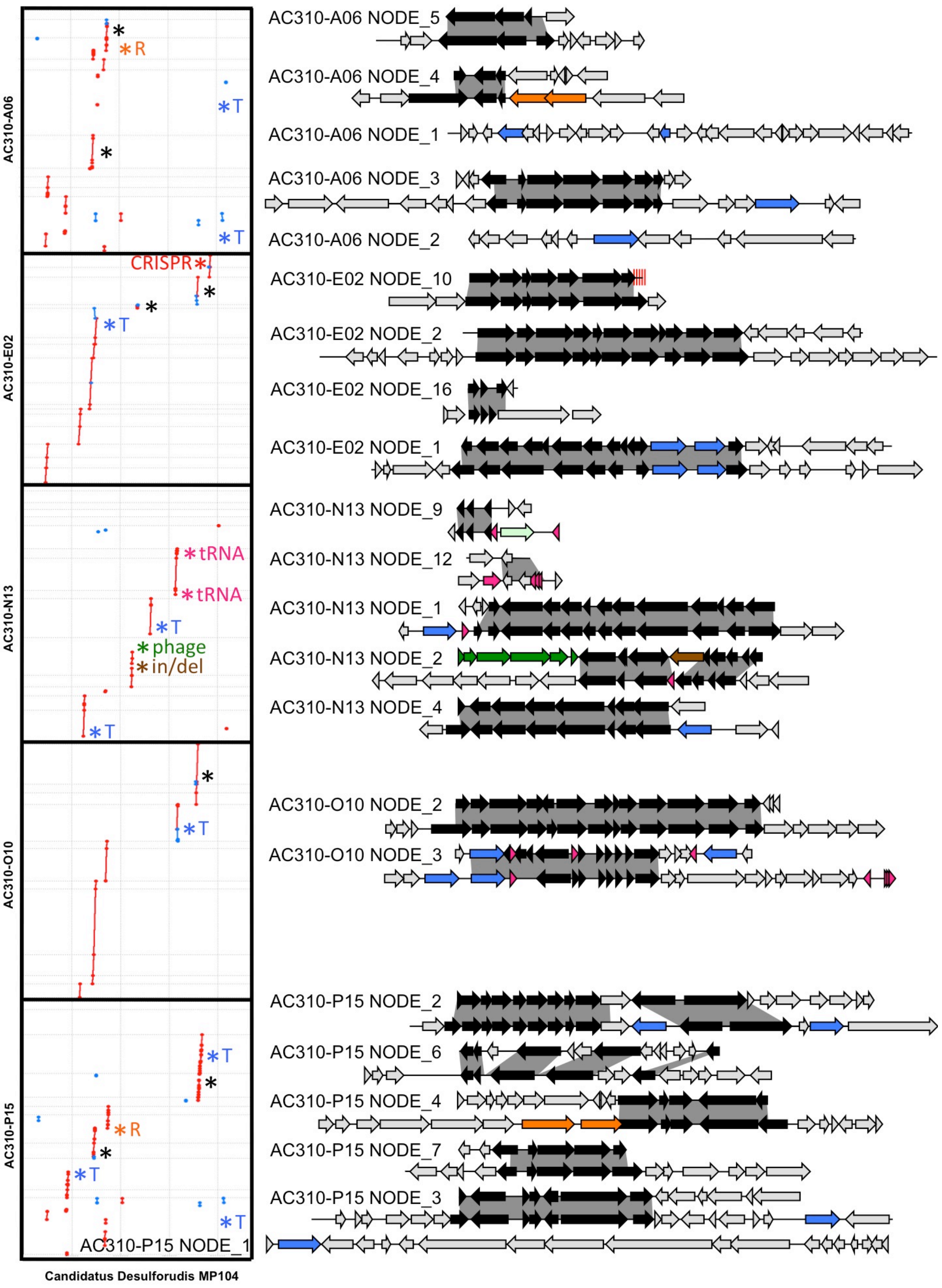


**Supplementary Figure 2:** Alignments of SAGs against *Ca. D. audaxviator* MP104C. Contigs (named as nodes) containing non-homologous regions (putative HGT events) are displayed on the right side, with the following coloring: transposases in blue, tRNAs in pink, recombinases in orange, insertions/deletions in brown, phages in dark green, CRISPR in red, integrase in light green, and unclassified in grey. Black arrows point to genes that are conserved in both the SAGs and *Ca. D. audaxviator* MP104C.

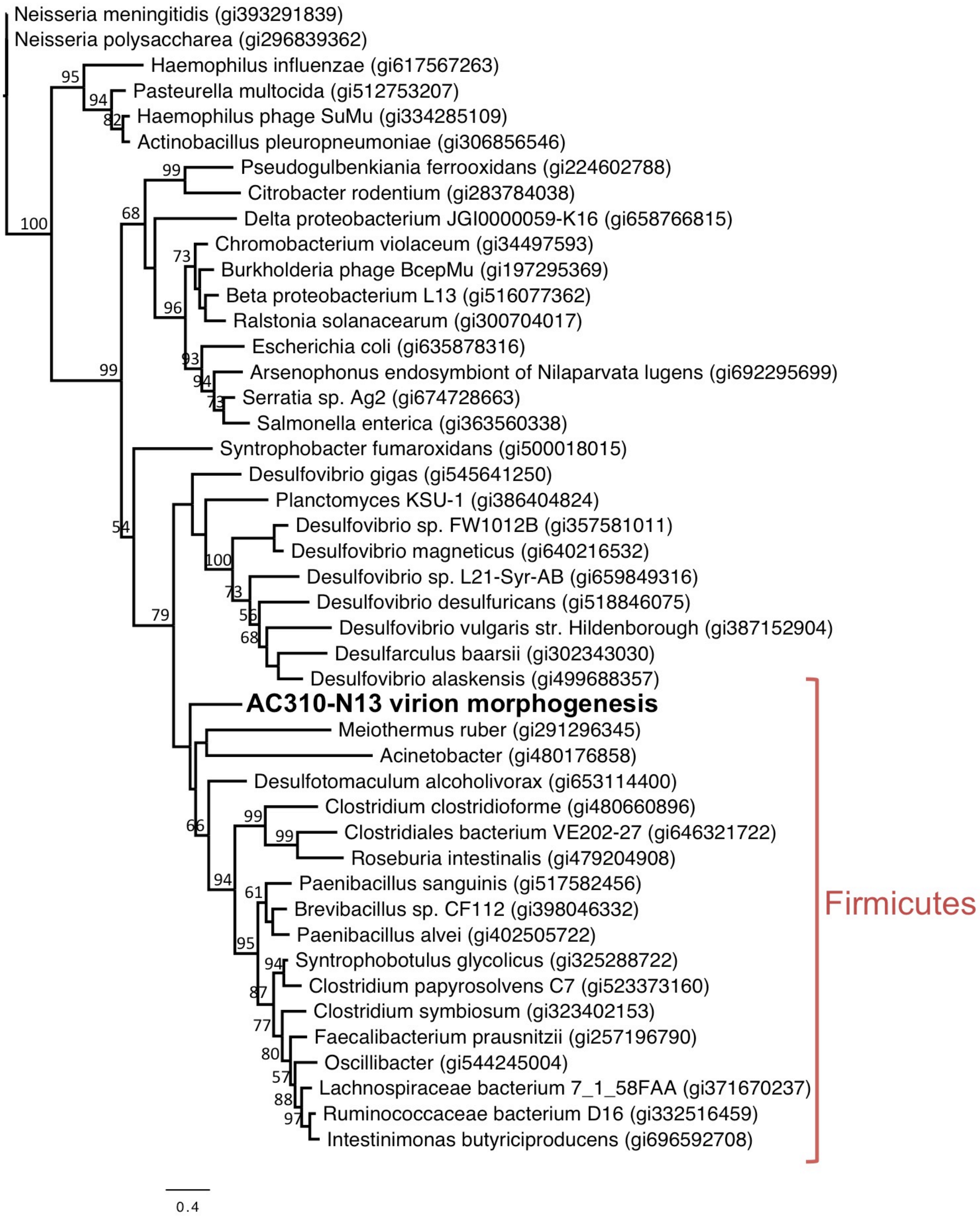


**Supplementary Figure 3:** Genetic relatedness of the virion morphogenesis protein of the transposable phage found in the SAG AC-310-N13 with other phages found in Firmicutes and Proteobacteria [maximum likelihood; 100 bootstrap replicates; WAG model with a gamma distribution (+G), estimated rates of variation among sites and a proportion of invariable sites (+I)].

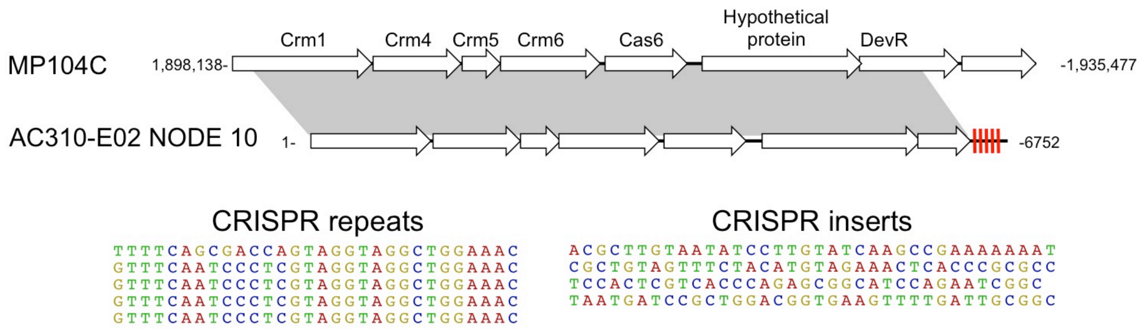


**Supplementary Figure 4:** Comparison of a pair of CRISPR loci found in AC-310-E02 and Ca. *D. audaxviator* MP104C showing differences within a repeat region (red bars).

**Supplementary Table 1:** Geographical, physical and chemical characteristics of the studied environmental samples. "N.A." = not available.

| Sample | Tau Tona, SAGs | Tau Tona, metagenome | Masimong, metagenome | Mponeng, metagenome |
|---|---|---|---|---|
| **Mine** | Tau Tona | Tau Tona | Masimong | Mponeng |
| **Borehole** | DPH5057 - TT109Bh1 | LIB107A - TT107 | LO478 - MM5 1940(46) | MP104 |
| **Date of collection** | January 19, 2012 | August 24, 2011 | July 20, 2012 | September 27, 2002 |
| **Rock formation** | Witwatersrand quartzite | Witwatersrand quartzite | Witwatersrand quartzite | Witwatersrand quartzite |
| **Latitude** | 26.417° S | 26.417° S | 27.976° S | 26.433° S |
| **Longitude** | 27.427° E | 27.427° E | 26.875° E | 27.433° E |
| **Mine elevation (MASL)** | 1,658.00 | 1,658 | 1,389 | 1,2 |
| **Depth (mbls)** | 3,136 | 3,048 | 1,900 | 2,8 |
| **Age (kyr)** | 16-21 | 1-6 | >40-80 | N.A. |
| **Temperature (ºC)** | 47.5 | 52.1 | 40.7 | >60 |
| **pH** | 8.87 | 8.6 | 8.18 | 9.3 |
| **Measured Eh (mV)** | 185 | N.A. | N.A. | -330 |
| **Conductivity (mS/cm)** | 0.49 | N.A. | N.A. | N.A. |
| **$O_2$ (ppm)** | 0.7 | N.A. | N.A. | N.A. |
| **Soluble Fe (ppm)** | <0.1 | 0.1 | N.A. | N.A. |
| **Reference** | This study | (Lau et al., 2014) | (Magnabosco et al., 2014) | (Lin et al., 2006) |

**Supplementary Table 2:** Metagenomic sequencing effort.

| Sample | Tau Tona | Masimong | Mponeng |
|---|---|---|---|
| **Sequencing method** | Illumina | Illumina | 454 (+ Sanger) |
| **Total number of reads** | 17,616,121 | 10,578,667 | 518,272 (31,218) |
| **Median sequence length** | 157 bp | 159 bp | 109 bp (1,035 bp) |
| **Reference** | (Lau et al., 2014) | (Magnabosco et al., 2014) | (Chivian et al., 2008) |

**Supplementary Table 3:** Average nucleotide identity (ANI) among SAGs and Ca. *D. audaxviator* MP104C. NA: Not available, due to absence of homologous regions.

| | | MP104C | SAG AC-310 | | | | |
|---|---|---|---|---|---|---|---|
| | | | A06 | E02 | N13 | O10 | P15 |
| MP104C | | | 91.80 | 99.63 | 88.58 | 98.41 | 93.69 |
| AC-310 | A06 | 92.19 | --- | 91.08 | 96.13 | 97.62 | 99.99 |
| | E02 | 99.96 | 89.77 | --- | NA | 99.36 | 93.19 |
| | N13 | NA | 99.98 | NA | --- | 94.80 | NA |
| | O10 | 99.98 | 94.61 | 99.41 | 94.88 | --- | 94.82 |
| | P15 | 92.93 | 99.99 | 90.32 | NA | 95.97 | --- |

**Supplementary Table 4:** Genes found on SAG contigs with no homology to *D. audaxviator* MP104C.

| Contig ID | Gene IMG ID | Product Name | DNA Seq Length | AA Seq Length | COG Categories |
|---|---|---|---|---|---|
| AC-310-A06_NODE_10 | 2596837198 | hypothetical protein | 195 | 64 | |
| | 2596837199 | hypothetical protein | 162 | 53 | |
| | 2596837200 | hypothetical protein | 477 | 158 | |
| | 2596837201 | Cytotoxic translational repressor of toxin-antitoxin stability system | 375 | 124 | J D |
| | 2596837202 | hypothetical protein | 444 | 147 | |
| | 2596837203 | hypothetical protein | 228 | 75 | |
| AC-310-A06_NODE_8 | 2596837190 | hypothetical protein | 1230 | 409 | |
| | 2596837191 | hypothetical protein | 1293 | 431 | |
| AC-310-N13_NODE_10 | 2600314776 | hypothetical protein | 207 | 68 | |
| | 2600314777 | Predicted nucleic acid-binding protein, contains PIN domain | 531 | 176 | R |
| | 2600314778 | Integrase core domain | 798 | 265 | |
| | 2600314779 | Domain of unknown function (DUF3387) | 225 | 74 | |
| | 2600314780 | hypothetical protein | 204 | 67 | |
| AC-310-N13_NODE_11 | 2600314781 | hypothetical protein | 432 | 144 | |
| | 2600314782 | hypothetical protein | 483 | 160 | |
| | 2600314783 | Uncharacterized ATPase, putative transposase | 1020 | 339 | R |
| | 2600314784 | Mu transposase, C-terminal | 663 | 220 | |
| AC-310-N13_NODE_13 | 2600314790 | Mu-like prophage major head subunit gpT | 396 | 131 | |
| | 2600314791 | Mu-like prophage major head subunit gpT | 195 | 64 | |
| | 2600314792 | Head fiber protein | 519 | 172 | |
| | 2600314793 | Mu-like prophage I protein | 1140 | 379 | R |
| AC-310-N13_NODE_15 | 2600314794 | hypothetical protein | 615 | 204 | |
| | 2600314795 | FOG: EAL domain | 1623 | 540 | T |
| AC-310-N13_NODE_5 | 2600314742 | Membrane protein putatively involved in post-translational modification of the autoinducing quorum-sensing peptide | 648 | 215 | O T K |
| | 2600314743 | SurA N-terminal domain | 882 | 293 | |
| | 2600314744 | hypothetical protein | 543 | 180 | |

| | | | | | |
|---|---|---|---|---|---|
| | 2600314745 | hypothetical protein | 486 | 161 | |
| AC-310-N13_NODE_5 | 2600314746 | Transposase domain (DUF772) | 384 | 127 | |
| | 2600314747 | hypothetical protein | 609 | 202 | |
| | 2600314748 | ( miscRNA ) | 77 | | |
| | 2600314749 | Retron-type reverse transcriptase | 1152 | 383 | L |
| AC-310-N13_NODE_7 | 2600314757 | hypothetical protein | 300 | 99 | |
| | 2600314758 | Mor transcription activator family | 315 | 104 | |
| | 2600314759 | Protein of unknown function (DUF1018) | 423 | 140 | |
| | 2600314760 | Sulfatase-modifying factor enzyme 1 | 918 | 305 | |
| AC-310-N13_NODE_7 | 2600314761 | hypothetical protein | 240 | 79 | |
| | 2600314762 | hypothetical protein | 663 | 220 | |
| | 2600314763 | Antidote-toxin recognition MazE | 258 | 85 | |
| | 2600314764 | hypothetical protein | 216 | 71 | |
| AC-310-P15_NODE_10 | 2596837655 | hypothetical protein | 285 | 95 | |
| | 2596837656 | hypothetical protein | 345 | 114 | |
| | 2596837657 | Uncharacterized conserved protein | 1542 | 513 | S |
| | 2596837658 | hypothetical protein | 357 | 118 | |
| | 2596837659 | hypothetical protein | 354 | 117 | |
| AC-310-P15_NODE_5 | 2596837613 | hypothetical protein | 1014 | 337 | J |
| | 2596837614 | hypothetical protein | 795 | 264 | |
| | 2596837615 | hypothetical protein | 495 | 164 | |
| | 2596837616 | hypothetical protein | 3717 | 1238 | |
| | 2596837617 | hypothetical protein | 1128 | 375 | |
| | 2596837618 | AAA-like domain | 1074 | 357 | |
| | 2596837619 | hypothetical protein | 360 | 119 | |
| | 2596837620 | Reverse transcriptase (RNA-dependent DNA polymerase) | 759 | 252 | |
| | 2596837612 | hypothetical protein | 216 | 71 | |

**Supplementary Table 5:** Recruitment of metagenomic fragments by *D. audaxviator* MP104C and by SAGs. Provided are counts of recruited reads and, in parenthesis, percentages of recruited reads. The latter are estimates of recruitment by complete SAG genomes, assuming that a) all *D. audaxviator* genomes are equal to the length of MP104 (2,349,476 bp); and b) recruitment is evenly distributed over the length of a genome. Metagenomic recruitment was performed with BLASTn using a 95% identity threshold.

| Genome | Tau Tona | Masimong | Northam | Mponeng |
|---|---|---|---|---|
| MP104C | 2,256,006 (12.8%) | 59,476 (0.56%) | 42 (0.00%) | 480,303 (92.7%) |
| AC-310-A06 | 53,280 (10.6%) | 860 (0.29%) | 210 (0.14%) | 6,306 (42.9%) |
| AC-310-E02 | 158,023 (16.3%) | 4,121 (0.71%) | 604 (0.21%) | 34,871 (122.4%) |
| AC-310-N13 | 70,985 (12.1%) | 1,106 (0.31%) | 356 (0.21%) | 7,761 (44.9%) |
| AC-310-O10 | 87,819 (14.7%) | 2,914 (0.81%) | 345 (0.20%) | 22,945 (130.5%) |
| AC-310-P15 | 88,428 (10.9%) | 1,398 (0.29%) | 299 (0.13%) | 11,692 (49.1%) |

**Supplementary Table 6:** Genome content discrepancies between SAGs and *D. audaxviator* MP104C.

| SAG | # of genes | # of genes not found in MP104C | % of genes not found in MP104C |
|---|---:|---:|---:|
| AC-310-A06 | 84 | 24 | 28.5 |
| AC-310-E02 | 148 | 0 | 0.0 |
| AC-310-N13 | 105 | 34 | 32.3 |
| AC-310-O10 | 105 | 7 | 6.7 |
| AC-310-P15 | 119 | 38 | 31.9 |

**Supplementary Table 7:** Genes encoded on contigs that contain putative horizontal gene transfer events. Color legend: transposases in blue, tRNAs in pink, recombinases in orange, insertions/deletions in brown, phages in green, CRISPR in red. Genes that are shared by SAGs and MP104C are in grey.

| | IMG gene ID | Product Name | DNA Seq Length | COG Categories |
|---|---|---|---|---|
| AC-310-A06_NODE_1 | 2596837120 | YcfA-like protein | 264 | |
| | 2596837121 | Uncharacterized conserved protein | 375 | S |
| | 2596837122 | hypothetical protein | 345 | |
| | 2596837123 | Transposase DDE domain | 1041 | |
| | 2596837124 | hypothetical protein | 423 | |
| | 2596837125 | hypothetical protein | 186 | |
| | 2596837126 | LytTr DNA-binding domain | 312 | |
| | 2596837127 | Membrane protein putatively involved in post-translational modification of the autoinducing quorum-sensing peptide | 648 | O T K |
| | 2596837128 | hypothetical protein | 405 | |
| | 2596837129 | SurA N-terminal domain | 882 | |
| | 2596837130 | hypothetical protein | 543 | |
| | 2596837131 | hypothetical protein | 486 | |
| | 2596837132 | Transposase domain (DUF772) | 402 | |
| | 2596837133 | hypothetical protein | 609 | |
| | 2596837134 | hypothetical protein | 354 | |
| | 2596837135 | AAA domain | 516 | |
| | 2596837136 | Winged helix-turn helix/Mu transposase, C-terminal/Integrase core domain | 1335 | |
| | 2596837137 | hypothetical protein | 615 | |
| | 2596837138 | hypothetical protein | 126 | |
| | 2596837139 | hypothetical protein | 234 | |
| | 2596837140 | Protein of unknown function (DUF1670) | 582 | |
| | 2596837141 | Protein of unknown function (DUF1670) | 585 | |
| | 2596837142 | Protein of unknown function (DUF1670) | 1296 | |
| | 2596837143 | hypothetical protein | 237 | |

| | | | | |
|---|---|---|---|---|
| | 2596837144 | hypothetical protein | 873 | |
| | 2596837145 | hypothetical protein | 168 | |
| | 2596837146 | Membrane protein putatively involved in post-translational modification of the autoinducing quorum-sensing peptide | 654 | O K T |
| AC-310-A06_NODE_2 | 2596837147 | Binding-protein-dependent transport system inner membrane component | 432 | |
| | 2596837148 | molybdenum ABC transporter, periplasmic molybdate-binding protein | 792 | P |
| | 2596837149 | HMGL-like | 906 | |
| | 2596837150 | Uncharacterized protein conserved in bacteria | 411 | S |
| | 2596837151 | transcriptional regulator, AbrB family | 246 | K |
| | 2596837152 | Predicted nucleic acid-binding protein, contains PIN domain | 432 | R |
| | 2596837153 | transposase, IS4 family | 1719 | L |
| | 2596837154 | Predicted Fe-S oxidoreductases | 1269 | R |
| | 2596837155 | heat-inducible transcription repressor HrcA | 702 | |
| | 2596837156 | hypothetical protein | 555 | |
| | 2596837157 | DNA-directed RNA polymerase, beta subunit/140 kD subunit | 3426 | K |
| | 2596837158 | TIGR02688 family protein | 1155 | |
| AC-310-A06_NODE_3 | 2596837159 | hypothetical protein | 249 | |
| | 2596837160 | hypothetical protein | 267 | |
| | 2596837161 | hypothetical protein | 402 | |
| | 2596837162 | hypothetical protein | 987 | |
| | 2596837163 | hypothetical protein | 369 | |
| | 2596837164 | RecA-superfamily ATPases implicated in signal transduction | 1404 | T |
| | 2596837165 | diguanylate cyclase (GGDEF) domain | 1416 | |
| | 2596837166 | HDIG domain | 1179 | |
| | 2596837167 | Anti-anti-sigma regulatory factor (antagonist of anti-sigma factor) | 759 | T |
| | 2596837168 | Anti-anti-sigma regulatory factor (antagonist of anti-sigma factor) | 363 | T |
| | 2596837169 | Anti-sigma regulatory factor (Ser/Thr protein kinase) | 453 | T |

| | | | | |
|---|---|---|---|---|
| | 2596837170 | Stage II sporulation protein E (SpoIIE) | 621 | |
| AC-310-A06_NODE_4 | 2596837171 | HD domain | 489 | |
| | 2596837172 | diguanylate cyclase (GGDEF) domain | 864 | T |
| | 2596837173 | hypothetical protein | 372 | |
| | 2596837174 | Predicted ATPase with chaperone activity | 1530 | O |
| | 2596837175 | IstB-like ATP binding protein | 360 | |
| | 2596837176 | hypothetical protein | 198 | |
| | 2596837177 | EAL domain | 291 | |
| | 2596837178 | diguanylate cyclase (GGDEF) domain | 1182 | T |
| AC-310-A06_NODE_5 | 2596837179 | PAS domain S-box/diguanylate cyclase (GGDEF) domain | 1899 | T |
| | 2596837180 | Uncharacterized conserved protein | 1164 | S |
| | 2596837181 | hypothetical protein | 273 | |
| | 2596837182 | FOG: GGDEF domain | 1086 | T |
| AC-310-E02_NODE_1 | 2596837204 | Copper amine oxidase N-terminal domain | 411 | |
| | 2596837205 | Domain of unknown function (DUF4263) | 855 | |
| | 2596837206 | DNA methylase/Restriction endonuclease | 708 | |
| | 2596837207 | DNA methylase | 741 | |
| | 2596837208 | hypothetical protein | 204 | |
| | 2596837209 | Uncharacterized conserved protein | 1149 | S |
| | 2596837210 | Peptidase S24-like/Helix-turn-helix | 693 | |
| | 2596837211 | Nuclease-related domain | 558 | |
| | 2596837212 | hypothetical protein | 156 | |
| | 2596837213 | hypothetical protein | 153 | |
| | 2596837214 | Copper amine oxidase N-terminal domain | 537 | |
| | 2596837215 | transposase, IS605 OrfB family, central region | 1383 | |
| | 2596837216 | transposase, IS605 OrfB family, central region | 1146 | L |
| | 2596837217 | Helix-hairpin-helix motif | 576 | |
| | 2596837218 | hypothetical protein | 852 | |
| | 2596837219 | Ribbon-helix-helix domain | 330 | |
| | 2596837220 | hypothetical protein | 171 | |
| | 2596837221 | diguanylate cyclase (GGDEF) domain | 1707 | |
| | 2596837222 | diguanylate cyclase (GGDEF) domain | 795 | T |

| | | | | |
|---|---|---|---|---|
| | 2596837223 | Stage II sporulation protein E (SpoIIE) | 627 | |
| AC-310-E02_NODE_2 | 2596837224 | Undecaprenyl-phosphate galactose phosphotransferase, WbaP/exopolysaccharide biosynthesis polyprenyl glycosylphosphotransferase | 1449 | M |
| | 2596837225 | 3'-phosphoadenosine 5'-phosphosulfate sulfotransferase (PAPS reductase)/FAD synthetase and related enzymes | 687 | E H |
| | 2596837226 | Arylsulfatase A and related enzymes | 1452 | P |
| | 2596837227 | Acyl-[acyl carrier protein]--UDP-N-acetylglucosamine O-acyltransferase | 741 | M |
| | 2596837228 | dTDP-4-dehydrorhamnose 3,5-epimerase and related enzymes | 408 | M |
| | 2596837229 | Predicted pyridoxal phosphate-dependent enzyme apparently involved in regulation of cell wall biogenesis | 1122 | M |
| | 2596837230 | Glycosyltransferases involved in cell wall biogenesis | 1011 | M |
| | 2596837231 | Glycosyl transferase family 2 | 204 | |
| | 2596837232 | Methyltransferase domain | 636 | |
| | 2596837233 | Predicted glycosyltransferases | 1023 | R |
| | 2596837234 | hypothetical protein | 1101 | |
| | 2596837235 | dTDP-4-dehydrorhamnose 3,5-epimerase (EC 5.1.3.13) | 573 | M |
| | 2596837236 | Glycosyltransferase | 1149 | M |
| | 2596837237 | Methionine biosynthesis protein MetW | 642 | S |
| | 2596837238 | Glycosyltransferase | 1143 | M |
| | 2596837239 | hypothetical protein | 504 | |
| AC-310-E02_NODE_10 | 2596837308 | CRISPR-associated protein, Cmr1 family | 1170 | L |
| | 2596837309 | CRISPR-associated protein, Cmr4 family | 861 | L |
| | 2596837310 | CRISPR-associated protein, Cmr5 family | 378 | L |
| | 2596837311 | CRISPR-associated protein, Cmr6 family | 972 | |
| | 2596837312 | CRISPR-associated protein, Cas6 family | 795 | L |
| | 2596837313 | hypothetical protein | 1545 | |
| | 2596837314 | CRISPR-associated autoregulator DevR family | 522 | |

| | | | | |
|---|---|---|---|---|
| AC-310-E02_NODE_16 | 2596837344 | Soluble P-type ATPase | 471 | R |
| | 2596837345 | Late competence development protein ComFB | 279 | |
| | 2596837346 | Uncharacterised protein family (UPF0182) | 444 | |
| | 2596837347 | Sec-independent protein translocase protein (TatC) | 312 | |
| AC-310-N13_NODE_1 | 2600314691 | Type I restriction enzyme R protein N terminus (HSDR_N) | 408 | 135 |
| | 2600314692 | DUF218 domain | 342 | 113 |
| | 2600314693 | hypothetical protein | 189 | 62 |
| | 2600314694 | hypothetical protein | 429 | 142 |
| | 2600314695 | Succinate dehydrogenase/fumarate reductase, flavoprotein subunit | 1599 | 532 |
| | 2600314696 | 4Fe-4S dicluster domain/2Fe-2S iron-sulfur cluster binding domain | 684 | 227 |
| | 2600314697 | Heterodisulfide reductase, subunit B | 849 | 282 |
| | 2600314698 | 4Fe-4S dicluster domain | 381 | 126 |
| | 2600314699 | hydro-lyases, Fe-S type, tartrate/fumarate subfamily, beta region | 561 | 186 |
| | 2600314700 | fumarase, class I alpha subunit (EC 4.2.1.2) | 843 | 280 |
| | 2600314701 | TRAP transporter, 4TM/12TM fusion protein | 1989 | 662 |
| | 2600314702 | TRAP transporter solute receptor, TAXI family | 1038 | 345 |
| | 2600314703 | Response regulator of citrate/malate metabolism | 675 | 224 |
| | 2600314704 | Leucine rich repeat variant | 1080 | 360 |
| AC-310-N13_NODE_2 | 2600314705 | hypothetical protein | 273 | 90 |
| | 2600314706 | Protein of unknown function (DUF3486) | 531 | 176 |
| | 2600314707 | Mu-like prophage FluMu protein gp28 | 1299 | 432 |
| | 2600314708 | Mu-like prophage protein gp29 | 1536 | 511 |
| | 2600314709 | phage putative head morphogenesis protein, SPP1 gp7 family | 738 | 245 |
| | 2600314710 | hypothetical protein | 207 | 68 |
| | 2600314711 | inosine-5'-monophosphate dehydrogenase | 1458 | 485 |
| | 2600314712 | hypothetical protein | 234 | 77 |
| | 2600314713 | glutamate 2,3-aminomutase (EC 5.4.3.-) | 1263 | 420 |
| | 2600314714 | Uncharacterised protein | 1335 | 444 |

| | | | | |
|---|---|---|---|---|
| | | family (UPF0236) | | |
| | 2600314715 | phosphoglycerate mutase (EC 5.4.2.1) | 609 | 202 |
| | 2600314716 | Domain of unknown function (DUF3842) | 414 | 137 |
| | 2600314717 | hypothetical protein | 270 | 89 |
| | 2600314718 | Predicted metal-dependent phosphoesterases (PHP family) | 681 | 226 |
| AC-310-N13_NODE_4 | 2600314733 | PPIC-type PPIASE domain | 330 | 109 |
| | 2600314734 | Ku protein, prokaryotic | 852 | 283 |
| | 2600314735 | Uncharacterized conserved protein | 1218 | 405 |
| | 2600314736 | sporulation protein YhbH | 1155 | 384 |
| | 2600314737 | putative serine protein kinase, PrkA | 1974 | 657 |
| | 2600314738 | hypothetical protein | 123 | 40 |
| | 2600314739 | Predicted glutamine amidotransferase | 726 | 241 |
| | 2600314740 | UDP-N-acetylmuramyl tripeptide synthase | 1404 | 467 |
| | 2600314741 | Uncharacterised protein family (UPF0236) | 1308 | 435 |
| AC-310-N13_NODE_9 | 2600314770 | hypothetical protein | 282 | 93 |
| | 2600314771 | Gas vesicle protein | 231 | 76 |
| | 2600314772 | ( tRNA ) | 76 | |
| | 2600314773 | hypothetical protein | 186 | 61 |
| | 2600314774 | ( tRNA ) | 74 | |
| | 2600314775 | Xanthosine triphosphate pyrophosphatase | 606 | 201 |
| AC-310-N13_NODE_12 | 2600314785 | DNA-binding transcriptional activator of the SARP family | 1098 | 366 |
| | 2600314786 | hypothetical protein | 444 | 147 |
| | 2600314787 | ( tRNA ) | 76 | |
| | 2600314788 | ( tRNA ) | 76 | |
| | 2600314789 | ( tRNA ) | 77 | |
| AC-310-O10_NODE_2 | 2596837465 | Sugar transferases involved in lipopolysaccharide synthesis | 564 | M |
| | 2596837466 | 3'-phosphoadenosine 5'-phosphosulfate sulfotransferase (PAPS reductase)/FAD synthetase and related enzymes | 687 | E H |
| | 2596837467 | Arylsulfatase A and related enzymes | 1452 | P |
| | 2596837468 | Acyl-[acyl carrier protein]--UDP-N-acetylglucosamine O-acyltransferase | 741 | M |

| | | | | |
|---|---|---|---|---|
| | 2596837469 | hypothetical protein | 456 | |
| | 2596837470 | Predicted pyridoxal phosphate-dependent enzyme apparently involved in regulation of cell wall biogenesis | 1122 | M |
| | 2596837471 | hypothetical protein | 699 | |
| | 2596837472 | Glycosyl transferase family 2 | 204 | |
| | 2596837473 | Methyltransferase domain | 636 | |
| | 2596837474 | Predicted glycosyltransferases | 1023 | R |
| | 2596837475 | O-antigen ligase like membrane protein | 1419 | |
| | 2596837476 | Glycosyltransferase | 1143 | M |
| | 2596837477 | Methionine biosynthesis protein MetW | 612 | |
| | 2596837478 | Uncharacterized conserved protein | 231 | S |
| | 2596837479 | hypothetical protein | 168 | |
| | 2596837480 | hypothetical protein | 138 | |
| AC-310-O10_NODE_3 | 2596837481 | Ham1 family | 366 | |
| | 2596837482 | Transposase DDE domain | 1332 | |
| | 2596837483 | hypothetical protein | 177 | |
| | 2596837484 | ( tRNA ) | 74 | |
| | 2596837485 | hypothetical protein | 495 | |
| | 2596837486 | hypothetical protein | 168 | |
| | 2596837487 | Winged helix-turn helix/Integrase core domain | 1296 | L |
| | 2596837488 | ( tRNA ) | 76 | |
| | 2596837489 | hypothetical protein | 225 | |
| | 2596837490 | Gas vesicle protein | 231 | |
| | 2596837491 | Gas vesicle protein | 228 | |
| | 2596837492 | hypothetical protein | 300 | |
| | 2596837493 | Gas vesicle synthesis protein GvpO | 282 | |
| | 2596837494 | gas vesicle protein GvpN | 960 | |
| | 2596837495 | hypothetical protein | 528 | |
| | 2596837496 | hypothetical protein | 168 | |
| | 2596837497 | hypothetical protein | 495 | |
| | 2596837498 | ( tRNA ) | 74 | |
| | 2596837499 | Transposase DDE domain | 1332 | |
| | 2596837500 | Ham1 family | 366 | |
| AC-315-P15_NODE_2 | 2596837565 | hypothetical protein | 159 | |

| | | | | |
|---|---|---|---|---|
| | 2596837566 | RNA polymerase, sigma 28 subunit, SigD/FliA/WhiG | 774 | K |
| | 2596837567 | hypothetical protein | 438 | |
| | 2596837568 | flagellar hook-basal body protein | 741 | N |
| | 2596837569 | Chemotaxis protein; stimulates methylation of MCP proteins | 516 | N T |
| | 2596837570 | Methylase of chemotaxis methyl-accepting proteins | 810 | N T |
| | 2596837571 | Chemotaxis protein CheC, inhibitor of MCP methylation | 618 | N T |
| | 2596837572 | Response regulator receiver domain | 369 | |
| | 2596837573 | flagellar motor switch protein FliM | 999 | N |
| | 2596837574 | Chemotaxis protein CheC, inhibitor of MCP methylation | 1179 | N T |
| | 2596837575 | Predicted unusual protein kinase | 1674 | R |
| | 2596837576 | O-antigen ligase like membrane protein/Tetratricopeptide repeat | 2415 | |
| | 2596837577 | hypothetical protein | 186 | |
| | 2596837578 | 1-acyl-sn-glycerol-3-phosphate acyltransferase | 606 | I |
| | 2596837579 | Predicted esterase of the alpha-beta hydrolase superfamily | 936 | R |
| | 2596837580 | heat-inducible transcription repressor HrcA | 699 | |
| | 2596837581 | Response regulator receiver domain | 894 | |
| | 2596837582 | transcriptional regulator, AbrB family | 252 | K |
| | 2596837583 | Predicted nucleic acid-binding protein, contains PIN domain | 444 | R |
| AC-310-O10_NODE_3 | 2596837584 | ABC transporter | 309 | |
| | 2596837585 | [FeFe] hydrogenase, group B1/B3 | 1344 | R |
| | 2596837586 | Ribbon-helix-helix protein, copG family | 249 | |
| | 2596837587 | Predicted nucleic acid-binding protein, contains PIN domain | 426 | R |
| | 2596837588 | Amidases related to nicotinamidase | 552 | Q |
| | 2596837589 | PAS domain S-box/diguanylate cyclase (GGDEF) domain | 2562 | T |
| | 2596837590 | Uncharacterized conserved protein | 900 | S |
| | 2596837591 | Domain of unknown function | 504 | |

| | | | | |
|---|---|---|---|---|
| | | (DUF3368)/PIN domain | | |
| | 2596837592 | Predicted Zn peptidase | 1065 | E |
| | 2596837593 | Diadenosine tetraphosphate (Ap4A) hydrolase and other HIT family hydrolases | 372 | F G R |
| | 2596837594 | Protein of unknown function (DUF524) | 1326 | |
| | 2596837595 | hypothetical protein | 255 | |
| | 2596837596 | AAA domain (dynein-related subfamily) | 1857 | |
| AC-315-P15_NODE_4 | 2596837597 | HD domain | 318 | |
| | 2596837598 | Anti-anti-sigma regulatory factor (antagonist of anti-sigma factor) | 759 | T |
| | 2596837599 | Anti-anti-sigma regulatory factor (antagonist of anti-sigma factor) | 363 | T |
| | 2596837600 | Anti-sigma regulatory factor (Ser/Thr protein kinase) | 453 | T |
| | 2596837601 | Stage II sporulation protein E (SpoIIE) | 627 | |
| | 2596837602 | diguanylate cyclase (GGDEF) domain | 795 | T |
| | 2596837603 | diguanylate cyclase (GGDEF) domain | 1725 | |
| | 2596837604 | EAL domain | 291 | |
| | 2596837605 | hypothetical protein | 198 | |
| | 2596837606 | IstB-like ATP binding protein | 360 | |
| | 2596837607 | Mg chelatase-related protein | 1530 | O |
| | 2596837608 | hypothetical protein | 372 | |
| | 2596837609 | diguanylate cyclase (GGDEF) domain | 864 | T |
| | 2596837610 | PAS domain S-box/diguanylate cyclase (GGDEF) domain | 2241 | T |
| | 2596837611 | FIST C domain | 465 | |
| AC-315-P15_NODE_6 | 2596837621 | Predicted nucleic acid-binding protein, contains PIN domain | 504 | R |
| | 2596837622 | hypothetical protein | 321 | |
| | 2596837623 | Predicted nucleic-acid-binding protein, contains PIN domain | 447 | R |
| | 2596837624 | S-layer homology domain | 1743 | |
| | 2596837625 | hypothetical protein | 318 | |
| | 2596837626 | Nucleotidyl transferase of unknown function (DUF1814) | 720 | |
| | 2596837627 | Transglycosylase SLT domain/Tetratricopeptide repeat | 1812 | |
| | 2596837628 | Transposase IS66 family | 459 | |

| | | | | |
|---|---|---|---|---|
| | 2596837629 | IS66 Orf2 like protein | 240 | |
| | 2596837630 | hypothetical protein | 177 | |
| | 2596837631 | hypothetical protein | 507 | |
| | 2596837632 | hypothetical protein | 330 | |
| | 2596837633 | hypothetical protein | 498 | |
| AC-315-P15_NODE_7 | 2596837634 | GAF domain | 483 | |
| | 2596837635 | hypothetical protein | 402 | |
| | 2596837636 | hypothetical protein | 987 | |
| | 2596837637 | hypothetical protein | 369 | |
| | 2596837638 | RecA-superfamily ATPases implicated in signal transduction | 1404 | T |
| | 2596837639 | diguanylate cyclase (GGDEF) domain | 1416 | |
| | 2596837640 | GAF domain | 483 | |

**Supplementary Table 8:** Transposases encoded by the five *D. audaxviator* single amplified genomes. MGE = Mobile genetic element

| Contig ID | IMG gene ID | Length | ACLAME MGE ID | MGE Name | MGE Type | MGE Hosts | E-value | GenBank Annotation |
|---|---|---|---|---|---|---|---|---|
| AC-310-A06_NODE_1 | 2596837123 | 346 | mge:1938 | pACRY01 | plasmid | Acidiphilium cryptum JF-5 | 7.00E-58 | transposase, IS4 family protein |
| AC-310-A06_NODE_1 | 2596837132 | 133 | mge:1935 | pXAUT01 | plasmid | Xanthobacter autotrophicus Py2 | 7.00E-24 | transposase IS4 family protein |
| AC-310-A06_NODE_1 | 2596837136 | 444 | mge:1141 | pRHL3 | plasmid | Rhodococcus sp. RHA1 | 2.00E-59 | probable transposase |
| AC-310-A06_NODE_2 | 2596837153 | 572 | mge:1933 | pACRY05 | plasmid | Acidiphilium cryptum JF-5 | 2.00E-72 | Transposase-like protein |
| AC-310-E02_NODE_1 | 2596837215 | 460 | mge:212 | ece1 | plasmid | Aquifex aeolicus VF5 | 5.00E-11 | hypothetical protein |
| AC-310-E02_NODE_1 | 2596837216 | 381 | mge:1813 | pMAQU01 | plasmid | Marinobacter aquaeolei VT8 | 2.00E-86 | transposase, IS605 OrfB family |
| AC-310-E02_NODE_6 | 2596837285 | 159 | mge:814 | pRHL1 | plasmid | Rhodococcus sp. RHA1 | 1.00E-22 | transposase |
| AC-310-E02_NODE_7 | 2596837295 | 215 | mge:814 | pRHL1 | plasmid | Rhodococcus sp. RHA1 | 4.00E-40 | probable transposase |
| AC-310-E02_NODE_8 | 2596837297 | | | | | | | |
| AC-310-E02_NODE_13 | 2596837333 | 523 | mge:1006 | megaplasmid | plasmid | Ralstonia eutropha JMP134 | 1.00E-104 | Transposase IS66 |
| AC-310-O10_NODE_1 | 2596837448 | 464 | mge:814 | pRHL1 | plasmid | Rhodococcus sp. RHA1 | 3.00E-80 | probable transposase |
| AC-310-N13_NODE_11 | 2600314784 | 478 | mge:460 | pLI100 | plasmid | Listeria innocua; Listeria innocua Clip11262 | 5.00E-09 | hypothetical protein |
| AC-310-N13_NODE_5 | 2600314746 | 339 | mge:460 | pLI100 | plasmid | Listeria innocua; Listeria innocua Clip11262 | 4.00E-08 | transposase IS4 family protein |
| AC-310-N13_NODE_11 | 2600314783 | 127 | mge:1935 | pXAUT01 | plasmid | Xanthobacter autotrophicus Py2 | 7.00E-24 | Hypothetical protein |

| | | | | | | | | |
|---|---|---|---|---|---|---|---|---|
| AC-310-O10_NODE_1 | 2596837459 | 523 | mge:1006 | megaplasmid | plasmid | Ralstonia eutropha JMP134 | 1.00E-104 | Transposase IS66 |
| AC-310-O10_NODE_3 | 2596837482 | | | | | | | |
| AC-310-O10_NODE_3 | 2596837499 | | | | | | | |
| AC-315-P15_NODE_1 | 2596837547 | 555 | mge:1933 | pACRY05 | plasmid | Acidiphilium cryptum JF-5 | 4.00E-72 | Transposase-like protein |
| AC-315-P15_NODE_6 | 2596837628 | 152 | mge:1897 | pSMED03 | plasmid | Sinorhizobium medicae WSM419 | 9.00E-11 | transposase IS66 |
| AC-315-P15_NODE_8 | 2596837641 | 264 | mge:494 | pCC7120gamma | plasmid | Nostoc sp. PCC 7120 | 2.00E-05 | hypothetical protein |

**Supplementary Table 9:** Genes found on contigs that encode transposases.

| Contig ID | Gene IMG ID | Product Name | DNA Seq Length | COGs | COG Categories |
|---|---|---|---|---|---|
| AC-310-A06_NODE_1 | 2596837120 | YcfA-like protein | 264 | | |
| | 2596837121 | Uncharacterized conserved protein | 375 | COG1598 | S |
| | 2596837122 | hypothetical protein | 345 | | |
| | 2596837123 | Transposase DDE domain | 1041 | | |
| | 2596837124 | hypothetical protein | 423 | | |
| | 2596837125 | hypothetical protein | 186 | | |
| | 2596837126 | LytTr DNA-binding domain | 312 | | |
| | 2596837127 | Membrane protein putatively involved in post-translational modification of the autoinducing quorum-sensing peptide | 648 | COG4512 | O T K |
| | 2596837128 | hypothetical protein | 405 | | |
| | 2596837129 | SurA N-terminal domain | 882 | | |
| | 2596837130 | hypothetical protein | 543 | | |
| | 2596837131 | hypothetical protein | 486 | | |
| | 2596837132 | Transposase domain (DUF772) | 402 | | |
| | 2596837133 | hypothetical protein | 609 | | |
| | 2596837134 | hypothetical protein | 354 | | |
| | 2596837135 | AAA domain | 516 | | |
| | 2596837136 | Winged helix-turn helix/Mu transposase, C-terminal/Integrase core domain | 1335 | | |
| | 2596837137 | hypothetical protein | 615 | | |
| | 2596837138 | hypothetical protein | 126 | | |
| | 2596837139 | hypothetical protein | 234 | | |
| | 2596837140 | Protein of unknown function (DUF1670) | 582 | | |
| | 2596837141 | Protein of unknown function (DUF1670) | 585 | | |
| | 2596837142 | Protein of unknown function (DUF1670) | 1296 | | |
| | 2596837143 | hypothetical protein | 237 | | |
| | 2596837144 | hypothetical protein | 873 | | |
| | 2596837145 | hypothetical protein | 168 | | |
| | 2596837146 | Membrane protein putatively involved in post-translational modification of the autoinducing quorum-sensing peptide | 654 | COG4512 | O K T |
| AC-310-A06_NODE_2 | 2596837147 | Binding-protein-dependent transport system inner membrane component | 432 | | |

| | | | | | |
|---|---|---|---|---|---|
| | 2596837148 | molybdenum ABC transporter, periplasmic molybdate-binding protein | 792 | COG0725 | P |
| | 2596837149 | HMGL-like | 906 | | |
| | 2596837150 | Uncharacterized protein conserved in bacteria | 411 | COG3742 | S |
| | 2596837151 | transcriptional regulator, AbrB family | 246 | COG2002 | K |
| | 2596837152 | Predicted nucleic acid-binding protein, contains PIN domain | 432 | COG4113 | R |
| | 2596837153 | transposase, IS4 family | 1719 | COG5421 | L |
| | 2596837154 | Predicted Fe-S oxidoreductases | 1269 | COG0535 | R |
| | 2596837155 | heat-inducible transcription repressor HrcA | 702 | | |
| | 2596837156 | hypothetical protein | 555 | | |
| | 2596837157 | DNA-directed RNA polymerase, beta subunit/140 kD subunit | 3426 | COG0085 | K |
| | 2596837158 | TIGR02688 family protein | 1155 | | |
| AC-310-E02_NODE_1 | 2596837204 | Copper amine oxidase N-terminal domain | 411 | | |
| | 2596837205 | Domain of unknown function (DUF4263) | 855 | | |
| | 2596837206 | DNA methylase/Restriction endonuclease | 708 | | |
| | 2596837207 | DNA methylase | 741 | | |
| | 2596837208 | hypothetical protein | 204 | | |
| | 2596837209 | Uncharacterized conserved protein | 1149 | COG4748 | S |
| | 2596837210 | Peptidase S24-like/Helix-turn-helix | 693 | | |
| | 2596837211 | Nuclease-related domain | 558 | | |
| | 2596837212 | hypothetical protein | 156 | | |
| | 2596837213 | hypothetical protein | 153 | | |
| | 2596837214 | Copper amine oxidase N-terminal domain | 537 | | |
| | 2596837215 | transposase, IS605 OrfB family, central region | 1383 | | |
| | 2596837216 | transposase, IS605 OrfB family, central region | 1146 | COG0675 | L |
| | 2596837217 | Helix-hairpin-helix motif | 576 | | |
| | 2596837218 | hypothetical protein | 852 | | |
| | 2596837219 | Ribbon-helix-helix domain | 330 | | |
| | 2596837220 | hypothetical protein | 171 | | |
| | 2596837221 | diguanylate cyclase (GGDEF) domain | 1707 | | |
| | 2596837222 | diguanylate cyclase (GGDEF) domain | 795 | COG2199 | T |
| | 2596837223 | Stage II sporulation protein E (SpoIIE) | 627 | | |
| AC-310- | 2596837283 | Adenine specific DNA | 2691 | COG2189 | L |

| | | | | | |
|---|---|---|---|---|---|
| E02_NODE_6 | | methylase Mod | | | |
| | 2596837284 | Type III restriction enzyme, res subunit | 3066 | | |
| | 2596837285 | Transposase domain (DUF772) | 480 | | |
| | 2596837286 | phosphoenolpyruvate synthase (EC 2.7.9.2) | 861 | | |
| AC-310-E02_NODE_7 | 2596837287 | hypothetical protein | 750 | | |
| | 2596837288 | hypothetical protein | 369 | | |
| | 2596837289 | RecA-superfamily ATPases implicated in signal transduction | 1404 | COG0467 | T |
| | 2596837290 | diguanylate cyclase (GGDEF) domain | 1416 | | |
| | 2596837291 | HDIG domain | 1179 | | |
| | 2596837292 | Anti-anti-sigma regulatory factor (antagonist of anti-sigma factor) | 759 | COG1366 | T |
| | 2596837293 | Anti-anti-sigma regulatory factor (antagonist of anti-sigma factor) | 363 | COG1366 | T |
| | 2596837294 | hypothetical protein | 192 | | |
| | 2596837295 | Transposase domain (DUF772) | 645 | | |
| AC-310-E02_NODE_8 | 2596837296 | LSU ribosomal protein L12P | 393 | COG0222 | J |
| | 2596837297 | Transposase DDE domain | 1332 | | |
| | 2596837298 | DNA-directed RNA polymerase subunit beta (EC 2.7.7.6) | 3552 | COG0085 | K |
| | 2596837299 | RNA polymerase Rpb1, domain 2/RNA polymerase Rpb1, domain 1 | 1425 | | |
| AC-310-E02_NODE_13 | 2596837331 | hypothetical protein | 537 | | |
| | 2596837332 | hypothetical protein | 594 | | |
| | 2596837333 | Transposase and inactivated derivatives | 1572 | COG3436 | L |
| | 2596837334 | IS66 Orf2 like protein | 357 | | |
| | 2596837335 | hypothetical protein | 306 | | |
| AC-310-N13_NODE_5 | 2600314742 | Membrane protein putatively involved in post-translational modification of the autoinducing quorum-sensing peptide | 648 | 215 | O T K |
| | 2600314743 | SurA N-terminal domain | 882 | 293 | |
| | 2600314744 | hypothetical protein | 543 | 180 | |
| | 2600314745 | hypothetical protein | 486 | 161 | |
| | 2600314746 | Transposase domain (DUF772) | 384 | 127 | |
| | 2600314747 | hypothetical protein | 609 | 202 | |
| | 2600314748 | ( miscRNA ) | 77 | | |
| | 2600314749 | Retron-type reverse transcriptase | 1152 | 383 | L |
| AC-310- | 2600314781 | hypothetical protein | 432 | 144 | |

| | | | | | |
|---|---|---|---|---|---|
| N13_NODE_11 | | | | | |
| | 2600314782 | hypothetical protein | 483 | 160 | |
| | 2600314783 | Uncharacterized ATPase, putative transposase | 1020 | 339 | R |
| | 2600314784 | Mu transposase, C-terminal | 663 | 220 | |
| AC-310-O10_NODE_1 | 2596837442 | RecA-superfamily ATPases implicated in signal transduction | 1404 | COG0467 | T |
| | 2596837443 | diguanylate cyclase (GGDEF) domain | 1416 | | |
| | 2596837444 | HDIG domain | 1179 | | |
| | 2596837445 | Anti-anti-sigma regulatory factor (antagonist of anti-sigma factor) | 759 | COG1366 | T |
| | 2596837446 | Anti-anti-sigma regulatory factor (antagonist of anti-sigma factor) | 363 | COG1366 | T |
| | 2596837447 | hypothetical protein | 192 | | |
| | 2596837448 | Transposase domain (DUF772)/Transposase DDE domain | 1395 | | |
| | 2596837449 | Stage II sporulation protein E (SpoIIE) | 627 | | |
| | 2596837450 | diguanylate cyclase (GGDEF) domain | 795 | COG2199 | T |
| | 2596837451 | diguanylate cyclase (GGDEF) domain | 1707 | | |
| | 2596837452 | hypothetical protein | 354 | | |
| | 2596837453 | hypothetical protein | 171 | | |
| | 2596837454 | Ribbon-helix-helix domain | 330 | | |
| | 2596837455 | hypothetical protein | 1014 | | |
| | 2596837456 | hypothetical protein | 267 | | |
| | 2596837457 | hypothetical protein | 306 | | |
| | 2596837458 | IS66 Orf2 like protein | 357 | | |
| | 2596837459 | Transposase and inactivated derivatives | 1572 | COG3436 | L |
| | 2596837460 | hypothetical protein | 594 | | |
| | 2596837461 | Copper amine oxidase N-terminal domain | 897 | | |
| | 2596837462 | Domain of unknown function (DUF4263) | 609 | | |
| | 2596837463 | Adenine specific DNA methylase Mod | 1638 | COG2189 | L |
| | 2596837464 | hypothetical protein | 204 | | |
| AC-310-O10_NODE_3 | 2596837481 | Ham1 family | 366 | | |
| | 2596837482 | Transposase DDE domain | 1332 | | |
| | 2596837483 | hypothetical protein | 177 | | |
| | 2596837484 | ( tRNA ) | 74 | | |
| | 2596837485 | hypothetical protein | 495 | | |

| | | | | | |
|---|---|---|---|---|---|
| | 2596837486 | hypothetical protein | 168 | | |
| | 2596837487 | Winged helix-turn helix/Integrase core domain | 1296 | COG3415 | L |
| | 2596837488 | ( tRNA ) | 76 | | |
| | 2596837489 | hypothetical protein | 225 | | |
| | 2596837490 | Gas vesicle protein | 231 | | |
| | 2596837491 | Gas vesicle protein | 228 | | |
| | 2596837492 | hypothetical protein | 300 | | |
| | 2596837493 | Gas vesicle synthesis protein GvpO | 282 | | |
| | 2596837494 | gas vesicle protein GvpN | 960 | | |
| | 2596837495 | hypothetical protein | 528 | | |
| | 2596837496 | hypothetical protein | 168 | | |
| | 2596837497 | hypothetical protein | 495 | | |
| | 2596837498 | ( tRNA ) | 74 | | |
| | 2596837499 | Transposase DDE domain | 1332 | | |
| | 2596837500 | Ham1 family | 366 | | |
| AC-315-P15_NODE_1 | 2596837546 | hypothetical protein | 204 | | |
| | 2596837547 | transposase, IS4 family | 1668 | COG5421 | L |
| | 2596837548 | Predicted Fe-S oxidoreductases | 1269 | COG0535 | R |
| | 2596837549 | heat-inducible transcription repressor HrcA | 702 | | |
| | 2596837550 | hypothetical protein | 339 | | |
| | 2596837551 | hypothetical protein | 555 | | |
| | 2596837552 | DNA-directed RNA polymerase, beta subunit/140 kD subunit | 3426 | COG0085 | K |
| | 2596837553 | TIGR02688 family protein | 1458 | | |
| | 2596837554 | hypothetical protein | 2295 | | |
| | 2596837555 | hypothetical protein | 4170 | | |
| | 2596837556 | Protein of unknown function (DUF4007) | 720 | | |
| | 2596837557 | 3'-phosphoadenosine 5'-phosphosulfate sulfotransferase (PAPS reductase)/FAD synthetase and related enzymes | 2301 | COG0175 | E H |
| | 2596837558 | hypothetical protein | 639 | | |
| | 2596837559 | Protein of unknown function, DUF488 | 435 | | |
| | 2596837560 | Protein of unknown function, DUF488 | 456 | | |
| | 2596837561 | hypothetical protein | 363 | | |
| | 2596837562 | Uncharacterized conserved protein | 453 | COG1598 | S |
| | 2596837563 | Predicted nucleic acid-binding protein, contains PIN domain | 432 | COG4113 | R |
| | 2596837564 | Protein of unknown function (DUF1778) | 237 | | |

| | | | | | |
|---|---|---|---|---|---|
| AC-315-P15_NODE_6 | 2596837621 | Predicted nucleic acid-binding protein, contains PIN domain | 504 | COG1487 | R |
| | 2596837622 | hypothetical protein | 321 | | |
| | 2596837623 | Predicted nucleic-acid-binding protein, contains PIN domain | 447 | COG5573 | R |
| | 2596837624 | S-layer homology domain | 1743 | | |
| | 2596837625 | hypothetical protein | 318 | | |
| | 2596837626 | Nucleotidyl transferase of unknown function (DUF1814) | 720 | | |
| | 2596837627 | Transglycosylase SLT domain/Tetratricopeptide repeat | 1812 | | |
| | 2596837628 | Transposase IS66 family | 459 | | |
| | 2596837629 | IS66 Orf2 like protein | 240 | | |
| | 2596837630 | hypothetical protein | 177 | | |
| | 2596837631 | hypothetical protein | 507 | | |
| | 2596837632 | hypothetical protein | 330 | | |
| | 2596837633 | hypothetical protein | 498 | | |
| AC-315-P15_NODE_8 | 2596837641 | conserved hypothetical protein (putative transposase or invertase) | 795 | | |
| | 2596837642 | hypothetical protein | 486 | | |
| | 2596837643 | Predicted nucleoside-diphosphate sugar epimerases | 1860 | COG1086 | M G |

## References


Chivian, D., Brodie, E., Alm, E., Culley, D., Dehal, P., DeSantis, T., Gihring, T., Lapidus, A., Lin, L.-H., Lowry, S., et al. (2008). Environmental genomics reveals a single-species ecosystem deep within Earth. *Science* 322, 275–278. doi:10.1126/science.1155495.

Lau, M. C. Y., Cameron, C., Magnabosco, C., Brown, C. T., Schilkey, F., Grim, S., Hendrickson, S., Pullin, M., Lollar, B. S., Heerden, E. Van, et al. (2014). Phylogeny and phylogeography of functional genes shared among seven terrestrial subsurface metagenomes reveal N-cycling and microbial evolutionary relationships. 5, 1–17. doi:10.3389/fmicb.2014.00531.

Lin, L.-H., Wang, P.-L., Rumble, D., Lippmann-Pipke, J., Boice, E., Pratt, L. M., Sherwood Lollar, B., Brodie, E. L., Hazen, T. C., Andersen, G. L., et al. (2006). Long-term sustainability of a high-energy, low-diversity crustal biome. *Science* 314, 479–482. doi:10.1126/science.1127376.

Magnabosco, C., Tekere, M., Lau, M. C. Y., Linage, B., Kuloyo, O., Erasmus, M., Cason, E., van Heerden, E., Borgonie, G., Kieft, T. L., et al. (2014). Comparisons of the composition and biogeographic distribution of the bacterial communities occupying South African thermal springs with those inhabiting deep subsurface fracture water. *Front. Microbiol.* 5, 679. doi:10.3389/fmicb.2014.00679.
